# The inconsistent effects of tDCS in rehabilitation and cognitive enhancement: sources of variability and paths to personalization

**DOI:** 10.3389/fnhum.2026.1817726

**Published:** 2026-06-11

**Authors:** Andrey Timashkov, Sergey Andreev, Anna Safonova, Samira Zangieva, Dzerassa Kadieva, Oksana Zinchenko

**Affiliations:** 1 Centre for Cognition and Decision Making, Institute for Cognitive Neuroscience, HSE University, Moscow, Russia; 2 Faculty of Social Sciences, School of Psychology, HSE University, Moscow, Russia

**Keywords:** Alzheimer’s disease, cognitive enhancement, inter-individual variability, Parkinson’s disease, rehabilitation, tDCS, transcranial direct current stimulation

## Abstract

**Background/aims:**

Transcranial direct current stimulation (tDCS) has emerged as a promising intervention in both rehabilitation and cognitive enhancement, yet its effects remain inconsistent across studies. This variability has raised questions about the underlying mechanisms influencing tDCS efficacy.

**Methods:**

In this review, we address the issue of the inconsistent effect of tDCS on motor and cognitive domains across studies in healthy individuals and those with neurodegenerative disorders. A review of literature in the field was conducted using PubMed and Google Scholar.

**Results:**

Research indicates that individual anatomical differences among subjects may contribute to the inconsistent outcomes observed, as variations in current density at targeted brain regions and genetic variations responsible for the stimulation effect. Understanding the factors that contribute to the inconsistent effects of tDCS will be essential for enhancing its application in clinical settings and maximizing its potential benefits in cognitive rehabilitation and enhancement.

**Conclusion:**

Future research should focus on optimizing tDCS parameters and exploring individualized approaches to treatment, taking into account the diverse responses observed in different populations.

## Introduction

Transcranial electrical stimulation (tES) encompasses several techniques that apply electric current to the scalp: transcranial direct current stimulation (tDCS) delivers a constant, low-intensity (1–2 mA) current; transcranial alternating current stimulation (tACS) uses a sinusoidal waveform; and transcranial random noise stimulation (tRNS) applies current with random fluctuations (0.1–640 Hz) (Antal et al., 2008; Nitsche and Paulus, 2001). Unlike transcranial magnetic stimulation (TMS), which induces suprathreshold neuronal firing, tES modulates resting membrane potentials at a subthreshold level and thus does not directly elicit action potentials (Barker et al., 1985; Nitsche and Paulus, 2000, 2001; Purpura and McMurtry, 1965). Depending on stimulation parameters, both modalities—including theta-burst TMS, repetitive TMS, and tDCS—can induce lasting facilitatory or inhibitory plasticity (Dayan et al., 2013; Nitsche and Paulus, 2001).

Furthermore, in addition to its application in cortical stimulation, tDCS has demonstrated considerable promise in the stimulation of subcortical regions of the brain, although mostly in animal models (Bolzoni et al., 2013; Nitsche and Paulus, 2000). It has also been found to induce plasticity mechanisms in the brain by enhancing synaptic strength (Polanía et al., 2011) and modulating the regulation of oxygen supply through control of blood flow (Zheng et al., 2011).

The simultaneous application of stimulation alongside behavioral tasks presents greater challenges when using repetitive transcranial magnetic stimulation (rTMS) compared to transcranial electrical stimulation (tES). This is because the suprathreshold activations associated with rTMS can potentially disrupt task-related neural activity, whereas the subthreshold polarization induced by tDCS enables online stimulation to modulate task-specific neuronal activation either by enhancing or reducing it.

While TMS exhibits higher spatial and temporal resolution, tES techniques, including tDCS are often characterized by their cost-effectiveness, ease of operation, and adaptability for double-blind, sham-controlled investigations (Rossini et al., 2015). Both techniques are highly valuable supplementary tools in the field of neuroscience research and possess the ability to address a fundamental constraint of neuroimaging techniques, namely the challenge of establishing causal involvement of brain regions or functional networks in distinct motor, perceptual, or cognitive processes.

The main obstacle to clinical adoption of tDCS is not safety or cost—it is variability. The same protocol, applied under identical conditions, can produce facilitatory, inhibitory, or null effects depending on who is being stimulated (López-Alonso et al., 2014; Wiethoff et al., 2014).

A narrative review by Vergallito et al. (2022) synthesized evidence specifically on intra-study inter-individual differences—a source of variability that, unlike between-study methodological heterogeneity, has historically been underaddressed in the field.

This variability is not random noise. It reflects a layered interaction between biological factors—individual neuroanatomy, cortical thickness, genetic polymorphisms—and methodological ones: electrode montage, current density, the participant’s baseline neurophysiological state. Without accounting for these sources of variability, reproducible tDCS protocols remain out of reach. This review synthesizes evidence on what drives that variability and describes emerging strategies for personalization, with particular focus on motor rehabilitation and neurodegenerative conditions.

## Methodology

This review is a narrative synthesis of literature on inter-individual variability in tDCS outcomes and protocol personalization. Searches were conducted in Google Scholar and PubMed, covering January 2000 to February 2026. Search terms, used individually and in combination, included: “transcranial direct current stimulation,” “tDCS variability,” “inter-individual differences tDCS,” “tDCS personalization,” “individualized tDCS protocol,” “finite element modeling tDCS,” “tDCS Alzheimer,” “tDCS Parkinson,” “tDCS cognitive enhancement,” “tDCS rehabilitation,” “tDCS genetics,” “tDCS responders,” and “tDCS anatomical factors.” Reference lists of identified articles, meta-analyses, and systematic reviews were hand-searched for additional sources.

Studies were included if they (1) examined or reported inter-individual variability in tDCS response, (2) investigated parameters affecting stimulation current distribution—anatomical, genetic, or technical—or (3) examined tDCS in motor rehabilitation or cognitive enhancement in healthy participants or those with neurodegenerative conditions. Studies focused primarily on other non-invasive brain stimulation methods were included only when directly relevant to tDCS mechanisms or comparisons. No formal PRISMA protocol was applied, consistent with the narrative review format; the search was nonetheless conducted systematically to reduce selection bias.

### Physiology of tDCS

tDCS is a non-invasive brain stimulation technique that involves the application of low-intensity electrical currents to specific areas of the scalp (Nitsche and Paulus, 2000). In contrast to alternative methods of neuromodulation, such as TMS, tDCS employs a consistent and low-intensity electrical current, typically ranging from 1 to 2 mA, in order to regulate the excitability of neurons (Bikson et al., 2016). This technique modulates the excitability of neurons in the targeted brain regions, thereby potentially influencing cognitive and motor functions (Thair et al., 2017). During tDCS, a small electrical current is administered by electrodes positioned on the scalp, typically comprising an anode (positive) and a cathode (negative). The electrical field produced by tDCS is hypothesized to elicit modifications in the resting membrane potentials of neurons, thereby affecting their excitability and subsequently impacting neural networks (Jackson et al., 2017; Khadka et al., 2020). Following a duration of tDCS targeting the motor cortices (M1), the excitability of the motor cortex was evaluated by means of single-pulse TMS applied over M1 to elicit motor evoked potentials (MEPs). Typically, it is believed that anodal stimulation of the primary motor cortex (M1) leads to an augmentation of cortical excitability, as evidenced by an increase in motor evoked potential (MEP) amplitude. A contrasting observation was made when cathodal stimulation was applied to the primary motor cortex (M1), resulting in a decrease in motor evoked potential (MEP) amplitude. The observed results in human subjects align with prior research conducted on animals about TES. These animal studies have previously documented polarity-dependent alterations in spontaneous brain activity following the application of a singular electric current (Jackson et al., 2017; Purpura and McMurtry, 1965). The bipolar electrode montage has gained widespread acceptance as the prevailing norm for tDCS research following the initial publication of Nitsche and Paulus’ seminal findings in human subjects. In the standard montage, tDCS is administered by polarizing two stimulating electrodes. In this context, it is observed that the anode, which serves as the destination, undergoes depolarization, while the cathode, which functions as the return path, experiences hyperpolarization. Consequently, an electric current is established, traveling from the cathode towards the anode. In general, tDCS has an initial monophasic waveform that gradually increases to reach the desired level of stimulation current. The duration of this acceleration phase may exhibit variability contingent upon the specific hardware employed for stimulation, although it generally persists for a duration of 30–60 s. The extended duration of the ramp-up period holds significance in mitigating scalp sensation that arises from the voltage alteration necessary to get the desired level of stimulation current. After reaching the desired goal current, the tDCS waveform is maintained at a constant level during the full stimulation phase, which normally lasts between 10 and 20 min, before gradually decreasing the current to 0 mA.

MRS studies have shown that anodal tDCS reduces GABA and raises glutamate/glutamine in the stimulated cortex; cathodal stimulation has the opposite effect (Stagg et al., 2009). This aligns with polarity-specific modulation of synaptic plasticity via NMDA receptor-dependent mechanisms—a neurochemical account of why tDCS shifts cortical excitability in the directions it does (Nitsche et al., 2003; Liebetanz, 2002).

### TDCS and neuroenhancement

Neuroenhancement refers to the use of techniques or interventions, such as tDCS, to improve cognitive abilities or enhance brain function beyond what is considered normal or typical. It involves the manipulation of brain activity in order to optimize performance in areas such as memory, attention, and problem-solving. Neuroenhancement has gained attention in various fields, including education, sports, and even in some medical applications. Up to date, numerous studies explored the application of tDCS in various fields of neuroenhancement, such as cognitive enhancement, clinical interventions, motor skill learning and neurorehabilitation.

TDCS, known for its safety, simplicity, portability, tolerability, and cost-effectiveness, has been extensively utilized in various applications. Researchers have conducted investigations into the possibility of tDCS in augmenting cognitive abilities, including memory, attention, and learning. Research findings have demonstrated encouraging outcomes in enhancing working memory and cognitive performance among healthy adults (Hoy et al., 2013; Andrews et al., 2011). Working memory is a prominent cognitive domain that has been extensively investigated in research on tDCS. Research findings indicate that the use of tDCS on the dorsolateral prefrontal cortex (DLPFC), a critical brain region involved in working memory, has been associated with enhancements in performance on working memory tasks (Nissim et al., 2017; Park et al., 2014; Caulfield et al., 2022). The enhancement of working memory is a topic of special interest for persons who are striving to improve their cognitive abilities in daily tasks that involve the temporary storage and manipulation of information. Research on attention has also been a central focus in tDCS studies. By selectively modulating attentional processes, particularly within the parietal cortex, tDCS has shown promise in enhancing attentional control and reducing distractibility, effects that appear to depend on individual oscillatory dynamics and may be especially relevant in aging populations (Kraft et al., 2024).

tDCS has demonstrated potential as a therapy modality for a range of neurological and psychiatric conditions, encompassing depression, schizophrenia, and chronic pain (Brunoni et al., 2012). The efficacy of tDCS in alleviating depression has been emphasized in studies conducted by Loo et al. (2012). The non-invasive modulation of neuronal activity renders tDCS a compelling choice for therapeutic intervention. tDCS also has demonstrated potential in the treatment of chronic pain through its ability to modulate pain perception and enhance overall quality of life. Antal et al. (2010) demonstrated that tDCS has the potential to alleviate pain in different types of chronic pain disorders. This finding suggests that tDCS could serve as a non-pharmacological method for managing pain.

The utilization of tDCS in the context of neurorehabilitation is currently a subject of extensive investigation, particularly in relation to stroke rehabilitation and traumatic brain injury (Fregni and Pascual-Leone, 2007). The application of tDCS to specific regions of the motor cortex has the potential to augment motor function and facilitate recovery in persons experiencing motor deficits. The prospective applications of tDCS in motor rehabilitation are widely recognized as highly promising. It has been employed in the rehabilitation of motor and speech impairments caused by stroke (Elsner et al., 2020; Wang et al., 2017), as well as in the treatment of Parkinson’s disease (Sánchez-Kuhn et al., 2017), cerebral palsy, cerebellar ataxia, multiple sclerosis (Vitório et al., 2019). Multiple studies have indicated that the capacity of tDCS to regulate cortical excitability and facilitate neuroplasticity grants the technique the capability to impact motor learning. Consequently, this influence on motor learning has been observed to enhance rehabilitation outcomes, particularly in areas such as gait balance and motor functions of the lower and upper limbs (Santos Ferreira et al., 2019; Veldema and Gharabaghi, 2022). Despite its potential, there are differing viewpoints regarding the effects of tDCS on cortical excitability. Santos Ferreira et al. (2019) propose that tDCS can induce both short-term and long-term effects on cortical excitability. However, other scholars argue that the effects of tDCS are only temporary and emphasize the need for additional research. Specifically, they suggest that further investigation is necessary to establish a consistent protocol that can effectively influence lasting effects on the motor cortex of individuals with impairments. The utilization of tDCS as a supplementary approach to conventional rehabilitation procedures holds significant promise due to its capacity to augment brain plasticity and facilitate recovery.

The study of tDCS and neuroenhancement has the potential to revolutionize how we approach learning and performance. Understanding the effects of these techniques can lead to the development of more effective educational strategies and training programs. Additionally, studying neuroenhancement can provide valuable insights into the mechanisms underlying brain function, which can have implications for treating cognitive disorders and improving overall brain health.

### TDCS application in neurodegenerative disorders

#### Alzheimer’s disease

Alzheimer’s disease (AD) involves progressive neurodegeneration across cholinergic and glutamatergic systems, with amyloid-beta plaques and tau tangles driving synaptic dysfunction and cortical atrophy. The rationale for tDCS in AD is that the technique can boost synaptic plasticity through NMDA receptor-dependent LTP-like mechanisms and upregulate BDNF—both of which are compromised in AD (Nitsche et al., 2003; Stagg et al., 2009).

The left DLPFC is the most common target, given its role in working memory and executive function networks (Chen et al., 2022). The temporal lobe—particularly the left temporoparietal junction and the cortex overlying the hippocampus—has attracted increasing interest, motivated by the centrality of hippocampal-entorhinal circuits in the episodic memory loss that defines early AD. Chen et al. (2022) found temporal lobe stimulation yielded better outcomes than prefrontal targeting, particularly for memory. The reason is probably straightforward: temporal electrodes are closer to hippocampal memory circuitry. But tDCS cannot directly stimulate subcortical structures at standard intensities—the effects propagate via cortico-hippocampal projections and depend on residual white matter integrity.

Current density optimization matters more in AD than in healthy populations. Cortical atrophy increases the distance between electrode and active grey matter, reducing the effective field at the target. FEM studies have shown that current density reaching the hippocampal region via scalp electrodes is substantially lower in AD patients than in healthy controls, which argues for subject-specific montage optimization in this group (Fertonani and Miniussi, 2017). High-current (2 mA) or extended-duration (20–30 min) protocols have been explored to compensate, though safety data for these parameters in cognitively impaired populations are still limited.

Combining tDCS with concurrent cognitive training has received increasing attention in AD. The idea is that behavioral engagement makes the stimulation task-relevant—it targets the circuits that are active during the task rather than at rest, potentially sharpening both the specificity and the magnitude of plasticity effects. Majdi et al. (2022) found significant improvements in global cognition (SMD = 1.640) and memory (SMD = 1.031) in AD patients, though attention measures showed less consistent benefit.

A more recent meta-analysis by Hou et al. (2024), which included 22 studies (1,074 patients) published up to February 2023, confirmed that tDCS significantly improved global cognition in MCI and AD patients across MMSE, MoCA, and MODA scoring scales. Dosage parameters, specifically current intensity and electrode size, significantly influenced outcomes. The authors noted that tDCS combined with other interventions showed superior effects to tDCS alone, supporting the additive role of concurrent behavioral engagement (Hou et al., 2024).

#### Parkinson’s disease

In Parkinson’s disease (PD) the pathophysiological target is different. PD involves degeneration of dopaminergic neurons in the substantia nigra pars compacta, which dysregulates the basal ganglia-thalamo-cortical motor circuit. The impairments in movement initiation, gait, and motor learning that result have motivated tDCS aimed at restoring cortical excitability and facilitating compensatory motor plasticity.

M1 and DLPFC are the most commonly targeted sites, with anodal stimulation intended to compensate for the reduced excitatory drive that follows dopaminergic denervation (Nguyen et al., 2024). The SMA’s role in gait initiation and bilateral motor coordination has also motivated SMA stimulation trials. The proposed mechanism goes beyond simple excitability enhancement: tDCS may also modulate the basal ganglia-cortex-cerebellum network through downstream connectivity effects, and there is some evidence it can trigger dopamine release from residual nigrostriatal terminals (Nguyen et al., 2024).

A 2022 systematic review and meta-analysis by Oliveira et al. found no significant effect of tDCS alone on motor function (UPDRS III), balance, gait, dyskinesias, or motor fluctuations in PD, regardless of brain area targeted. However, meta-regression identified that younger patients with milder symptoms showed small positive effects—suggesting that patient-level characteristics significantly moderate treatment response (Oliveira et al., 2022). This heterogeneity of response is consistent with the broader variability problem and reinforces the need for stratified trial design.

Dopaminergic medication state is a variable that most studies handle inconsistently. tDCS effects in the ON state (pharmacologically elevated dopamine) can differ substantially from effects in the OFF state—and can even be attenuated, consistent with the inverted-U model of dopamine-dependent plasticity. Medication timing therefore needs to be standardized in PD trials.

A 2024 systematic review and meta-analysis in NPJ Parkinson’s Disease included 38 studies and 12 randomized sham-controlled trials (263 PD patients) and found no significant overall differences between active and sham tDCS across motor function (UPDRS-III: SMD = −0.14, *p* = 0.74), gait, attention, executive function, or memory. Prediction intervals indicated substantial heterogeneity. Meta-regression showed small positive effects specifically in younger PD patients with milder baseline symptoms—a result that points to baseline disease severity as a moderator of tDCS efficacy and argues for disease-stage stratification in future trials (Duan and Zhang, 2024).

Future trials should incorporate FEM-guided montage planning and stratify by disease stage, medication state, and genotype - otherwise the same variability problem that plagues healthy-population tDCS research will persist here.

### Current limitations: sources of variability in tDCS outcomes

While the potential applications of tDCS are numerous, there are also several obstacles that hinder its effective implementation. Voarino et al. (2017) laid out several ethical problems specific to non-therapeutic tDCS use: coercion in competitive settings (academic, occupational), inequity of access, unknown long-term neurological risks, and the difficulty of meaningful informed consent when the mechanisms of action remain incompletely understood. The authors also pointed to a regulatory gap—consumer-grade tDCS devices are commercially available, yet existing neurotechnology frameworks have not caught up. In addition, the limited understanding of acceptability in mental healthcare as noted by Smits et al. (2021) and the limited understanding of acceptability in mental healthcare as noted by Smits et al. (2021) and Stagg and Nitsche (2011) have argued that the generalizability of findings in tDCS research is constrained. This assertion is based on their observation that the majority of tDCS studies have focused on the motor cortex. According to Polanía et al. (2011), there is a suggestion that tDCS has restricted spatial precision, perhaps resulting in the modulation of functional connectivity in brain regions beyond the electrode coverage. In a similar vein, Meinzer et al. (2012) believe that the approach has the potential to inadvertently affect connectivity within neural networks outside the range of electrode coverage.

The majority of research findings indicate that anodal tDCS has a positive effect on cortical excitability, as demonstrated by studies conducted by Bastani and Jaberzadeh (2012) and Horvath et al. (2015). Nevertheless, the observed impact was discovered to exhibit significant variability (López-Alonso et al., 2014; Wiethoff et al., 2014). Specifically, the study conducted by the López-Alonso et al. (2014) revealed that merely 12.5% of the entire sample exhibited the anticipated response to three distinct protocols of facilitative stimulation, encompassing repetitive transcranial magnetic stimulation (rTMS), anodal electrical stimulation (anodal tDCS), and paired transcranial magnetic stimulation (TMS). Furthermore, 25% of participants displayed a negative response to all three protocols, indicating an absence of noteworthy alterations in motor response. In a study conducted by the Wiethoff group in 2014, it was found that when testing the protocol of anodal and cathodal electrical stimulation on the same sample, there was considerable variability in the responses observed. Specifically, only 36% of participants demonstrated a “conventional” response, which involved facilitation of motor potential after anodal stimulation of the motor cortex and inhibition after cathodal stimulation. Conversely, 21% of participants exhibited an opposite effect, where inhibition occurred after anodal stimulation and facilitation occurred after cathodal stimulation. Additionally, 38% of participants showed potential facilitation after both protocols, while 5% showed inhibition after both protocols. Previous studies have demonstrated inconsistent and inadequately replicable findings (Vannorsdall et al., 2016; Buch et al., 2017) (see Table 1).

**Table 1:** Studies reporting inter-individual variability in tDCS outcomes.

Study	N	Population	Protocol	Key finding	Notes
López-Alonso et al. (2014)	56	Healthy adults	a-tDCS, rTMS, TMS	Only 12.5% showed expected facilitation across all three protocols; 25% showed suppression	Motor cortex; MEP
Wiethoff et al. (2014)	53	Healthy adults	a-tDCS, c-tDCS	36% conventional response; 21% reversed polarity effects	Motor cortex; MEP
	40	Healthy adults	a-tDCS (1 mA, 20 min)	High test–retest variability; individual response not predicted by group parameters	Reproducibility
Albizu et al. (2020)	30	Healthy adults	a-tDCS (1 mA)	ML + FEM model predicted motor response, AUC ~ 0.80	Machine learning + FEM
Cheeran et al. (2008)	52	Healthy adults	a-tDCS, theta-burst TMS	BDNF Val66Met Met-carriers showed attenuated or absent plasticity after tDCS	Genetic (BDNF)
Plewnia et al. (2015)	46	Healthy adults	a-tDCS over DLPFC	COMT Val158Met predicts cognitive response direction: Val/Val benefit, Met/Met no effect	Genetic (COMT)
Opitz et al. (2016)	14	Healthy adults	FEM (no active tDCS)	~50% of grey matter EF variance explained by 4 anatomical parameters	Anatomical FEM
Vergallito et al. (2022)	Review	Mixed	Review	~50% participants are consistent responders; variability driven by stable, variable, and contextual factors	Narrative review; PMID 35624908
Evans et al. (2022)	Review/model	Healthy adults	FEM	50–150% inter-individual variability in current direction across common montages	FEM; doi 10.1016/j.neuroimage.2022.119501
Razza et al. (2024)	40	Healthy adults	Prefrontal tDCS 1.5/3 mA	Individual E-field in DLPFC and ACC predicts working memory response; significant only at 3 mA	Cortex; PMID 38157837
Puri et al. (2015)	Review + older adults	Healthy older adults	a-tDCS 10 vs. 20 min	BDNF Val66Met significantly associated with inter- and intra-individual variability; duration-dependent effects	Cognitive Affective BN; PMID 36385251

### Anatomical factors and finite element modeling

Individual neuroanatomy is among the best-documented sources of tDCS variability. Current delivered to the scalp must pass through skin, subcutaneous fat, skull, cerebrospinal fluid (CSF), and brain parenchyma - layers that differ substantially in conductivity and, more importantly, in thickness and geometry from one person to the next. Those differences directly alter where the induced electric field ends up and how strong it is at the intended cortical target.

Low spatial precision is a primary reason for inconsistent tDCS outcomes. Finite element method (FEM) modeling using MRI-derived head geometry has clarified how much individual anatomy shapes current distribution. Opitz et al. (2016) showed that roughly 50% of the variance in cortical grey matter electric field strength can be explained by just four anatomical parameters: CSF layer thickness, skull thickness, gyral depth, and electrode-to-target distance. The assumption of uniform current delivery across participants - built into standard tDCS protocols—does not hold.

Subsequent work has further characterized the practical consequences. Evans et al. (2022) found substantial inter-individual variability in the direction - not just the magnitude—of the induced current across commonly used tDCS montages, with variability of 50–150% across participants. This means that even when field strength is nominally comparable, the neuronal populations being activated may differ systematically between individuals (Evans et al., 2022).

Each anatomical layer contributes differently. Skull defects and anomalies concentrate current locally (Datta et al., 2010). Subcutaneous fat changes scalp resistance and therefore affects how much current enters the head (Truong et al., 2013). The CSF layer acts as a low-resistance shunt—current preferentially flows through it rather than into cortex, reducing the dose reaching neural tissue (Opitz et al., 2016). Gyral orientation relative to the electrode determines whether the induced field is predominantly tangential or perpendicular to the cortical surface, which matters for which neurons are activated and in which direction (Miranda et al., 2013; Neuling et al., 2012; Sadleir et al., 2010).

Electrode geometry adds another layer of complexity. Currents are strongest beneath electrode edges, and particularly under edges closest to the return electrode (Miranda et al., 2006; Wagner et al., 2007). Maximum cortical stimulation may not occur under the center of the electrode but in cortical regions between electrodes (Miranda et al., 2013; Opitz et al., 2016). Electrode area and inter-electrode distance shape current spread and focality in ways that are nonetheless rarely modeled in practice (Faria et al., 2011).

Technical aspects of electrode placement that are routinely overlooked make the picture worse. Niemann et al. (2024) demonstrated in 240 datasets that deviations from planned electrode positions reduce current flow to target regions by 26–30% for focal tDCS set-ups—a finding with direct consequences for any study relying on focal montages without position verification (Niemann et al., 2024). The authors called for routine electrode position verification before and after stimulation as a minimum methodological standard.

Technical aspects of electrode construction that go unreported in most papers can also drive systematic differences between studies. These include rubber pad and gel layer thickness, gel conductivity, and the position of the cable connector on the electrode. Lower gel conductivity—around 1 S/m—appears preferable for limiting current spread at the electrode-skin interface (Saturnino et al., 2015). Rubber electrodes with conductive gel have replaced sponge electrodes in many settings, improving positional stability, but the widespread use of high-conductivity EEG gel introduces variability that is rarely acknowledged.

Most protocols position electrodes using the international 10–10 or 10–20 EEG systems and fix them with neoprene caps. This is convenient, but it has known problems: EEG landmarks do not reliably correspond to underlying cortical targets across individuals, cap-based systems offer limited focal targeting, and electrode slip during stimulation is common.

The clinical implications for stroke rehabilitation were demonstrated by van der Cruijsen et al. (2022), who showed that individualizing electrode montage in chronic stroke patients—using MRI-based FEM models—produced a 20% higher electric field at the motor target on average, with individual improvements reaching 52%, compared to standard montages (van der Cruijsen et al., 2022). This establishes a direct proof-of-concept that subject-specific optimization produces clinically meaningful differences in current delivery.

Albizu et al. (2020) showed that machine learning applied to FEM-derived electric field maps can predict individual tDCS response. More recently, Van Hoornweder et al. (2022) introduced the 2-SPED (2-sample prospective E-field dosing) approach, using E-field strength from a first population to individualize stimulation intensity in a second population across 300 participants, demonstrating that prospective E-field dosing can substantially reduce variability at target sites (Van Hoornweder et al., 2022). That individualization becomes especially critical in patients with brain atrophy: Bhattacharjee et al. (2024) showed that dementia patients with significant cortical atrophy require substantially higher personalized doses for the same target field strength, and that HD-tDCS with fixed electrode positions exceeded safe current limits (>4 mA) in 46% of those patients (Bhattacharjee et al., 2024).

### Genetic factors

Genetic variation is a second major source of inconsistency in tDCS response. The best-characterized case involves the Val66Met single nucleotide polymorphism in the BDNF gene. BDNF is involved in the LTP-like mechanisms thought to underlie tDCS after-effects (Antal et al., 2010). Met allele carriers (Val66Met or Met66Met) show attenuated or reversed motor cortex excitability changes compared to Val/Val homozygotes (Cheeran et al., 2008; Fritsch et al., 2010). The likely reason is impaired activity-dependent BDNF secretion in Met carriers—a process needed for consolidating tDCS-induced plasticity.

The interaction between BDNF genotype and stimulation duration is particularly relevant for clinical applications. Puri et al. (2015) examined 54 healthy older adults in a crossover study of 10- versus 20-min anodal tDCS over M1, and found that both inter-individual and intra-individual variability were significantly associated with BDNF Val66Met genotype. Met carriers showed distinctly different plasticity profiles depending on stimulation duration, with 20-min but not 10-min stimulation revealing genotype-dependent effects (Puri et al., 2015). These findings have direct implications for protocol design in aging populations, where BDNF-related plasticity changes are already reduced.

The Val158Met polymorphism in the COMT gene modulates prefrontal dopaminergic tone and predicts differential cognitive responses to DLPFC stimulation (Plewnia et al., 2015). Under the inverted-U model of dopamine, the optimal neuromodulatory effect depends on baseline dopamine levels - which COMT genotype partly determines. This may explain why the same DLPFC protocol enhances working memory in some participants and has no effect, or a negative one, in others.

A clinical demonstration of this genotype-tDCS interaction was provided by a 2024 study of post-stroke cognitive impairment, which found that COMT Val158Met genotype had a significant main effect on cognitive improvement following prefrontal anodal tDCS (2 mA, 10 sessions over DLPFC), while BDNF Val66Met did not show a significant interaction in this stroke sample. These findings highlight that the genetic predictors of tDCS response may differ across patient populations and stimulation targets (Ai et al., 2024).

Polymorphisms in the serotonin transporter gene (SLC6A4) have also been linked to variability in tDCS effects on mood and depression-related outcomes (Brunoni et al., 2013). The practical implication is that genotyping—at minimum for BDNF Val66Met and COMT Val158Met - could work as a stratification tool in tDCS research and, eventually, clinical practice. Including genotype data in tDCS trials is still uncommon, but is increasingly pushed as a prerequisite for reproducibility and precision stimulation protocols.

### Sensory factors and responder classification

A narrative review by Vergallito et al. (2022) provided the most comprehensive synthesis to date of intra-study inter-individual differences in tDCS effects, distinguishing between stable factors (anatomy, genetics), variable factors (baseline state, baseline performance), and contextual factors (task type, timing). The authors reviewed evidence from TMS-EEG studies and found that the proportion of responders across conditions typically falls around 50%, with consistent non-responders present across most studied protocols (Vergallito et al., 2022).

Nejadgholi et al. (2015) showed these two groups can be distinguished by differences in scalp voltage distribution between anode and cathode during stimulation—suggesting that objective, real-time biomarkers of individual stimulation delivery are within reach.

Machine learning approaches have been applied to this classification problem. Albizu et al. (2020) found that combining individualized electric field estimates with baseline neurophysiological data predicted motor cortex excitability changes after tDCS with AUC around 0.80.

Razza et al. (2024) investigated the relationship between individually simulated electric fields and working memory performance under prefrontal tDCS (1.5 mA and 3 mA) in 40 participants, finding that effects were significant only for the 3 mA group and only for emotional working memory tasks. Individual E-field magnitude in the DLPFC and anterior cingulate cortex - not the scalp dose - predicted behavioral outcomes, providing direct evidence that individual anatomical differences in E-field distribution drive cognitive variability under tDCS (Razza et al., 2024).

The brain’s neurophysiological state at stimulation onset—spontaneous oscillatory activity, excitation-inhibition balance, arousal—shapes tDCS response substantially (Silvanto and Pascual-Leone, 2008). Stimulation timing relative to task performance (online vs. offline) is therefore a biologically meaningful variable, not just a procedural detail.

### Neurophysiological factors

Even when anatomical, genetic, and sensory factors are accounted for, the brain’s functional state at the moment of stimulation introduces a further layer of variability. The effect of tDCS depends substantially on the baseline excitability of the targeted cortex, often described as the excitation–inhibition (E/I) balance (Silvanto and Pascual-Leone, 2008). If cortical excitability is already high, anodal stimulation may produce weaker or even reversed facilitation compared to when it is lower. This state-dependency means that identical tDCS parameters can yield different outcomes depending on whether the participant is at rest, engaged in a cognitive task, or in a particular phase of spontaneous oscillatory activity.

Ongoing EEG rhythms are a key determinant of state-dependency. Studies using real-time EEG have shown that the phase and power of sensorimotor oscillations (e.g., mu-rhythm) influence whether transcranial stimulation induces long-term potentiation- or depression-like plasticity (Zrenner et al., 2018). Building on these findings, closed-loop stimulation systems are being developed that trigger tDCS pulses only when the brain is in a “receptive” state, thereby reducing variability related to spontaneous fluctuations in cortical excitability (see also Paths to Personalization). Thus, neurophysiological state is both a source of variability and a target for individualized intervention.

### Methodological factors: stimulation parameters

Current density, intensity divided by electrode area, is among the main determinants of cortical field strength, yet it is reported inconsistently and varies widely between studies. Standard protocols use 1–2 mA through 25–35 cm2 electrodes, but high-definition tDCS configurations with smaller electrodes produce markedly different current distributions at nominally similar intensities (Monte-Silva et al., 2013).

Stimulation duration shows a non-linear dose–response relationship: too long a protocol can reverse the expected polarity-specific effect (Monte-Silva et al., 2013). Inter-session interval, session number, and concurrent behavioral engagement each interact with duration-dependent effects in ways that have not been systematically characterized across clinical populations.

The meta-analysis by Indahlastari et al. (2021) showed how methodological heterogeneity limits quantitative synthesis. The case for standardized, pre-registered protocols and pre-study computational modeling of electrode placement is hard to argue against.

**Figure 1:**
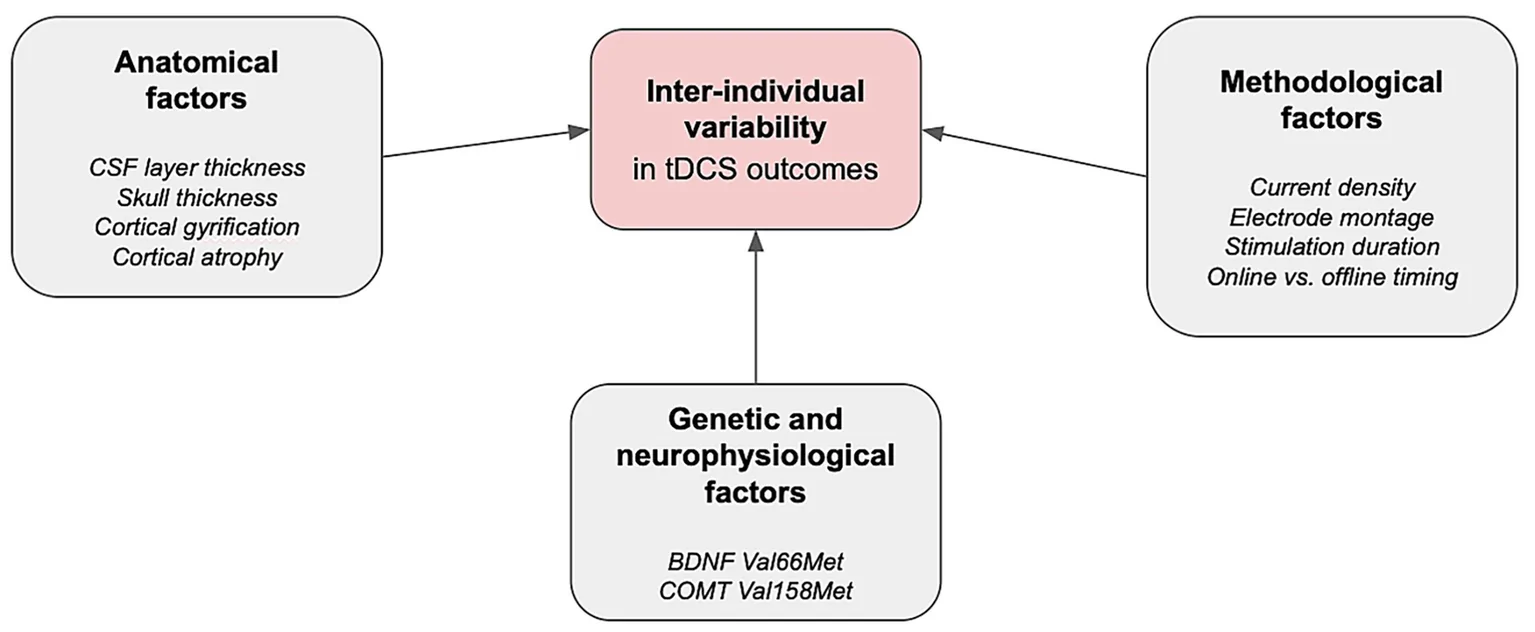
Principal sources of inter-individual variability in tDCS outcomes.

Table 2 summarizes the main factors that contribute to inter-individual variability in tDCS outcomes across anatomical, genetic, neurophysiological, and methodological categories, along with representative references (see Figure 1).

**Table 2:** Key factors contributing to inter-individual variability in tDCS outcomes.

Category	Factor	Direction of effect	Key references
Anatomical	CSF layer thickness	Thicker CSF shunts current; reduces cortical EF	Opitz et al. (2016) and Datta et al. (2010)
	Skull thickness	Greater thickness attenuates transcranial current	Opitz et al. (2016)
	Cortical gyrification	Gyral orientation relative to E-field changes neuronal activation direction	Miranda et al. (2013) and Evans et al. (2022)
	Cortical atrophy (AD/PD)	Increases electrode-to-target distance; reduces effective dose	Bhattacharjee et al. (2024) and Fertonani and Miniussi (2017)
	Electrode positioning error	26–30% reduction in target current for focal set-ups	Niemann et al. (2024)
Genetic	BDNF Val66Met	Met allele attenuates or reverses LTP-like plasticity; duration-dependent effects in older adults	Cheeran et al. (2008) and Puri et al. (2015)
	COMT Val158Met	Met allele alters prefrontal dopamine; changes DLPFC-tDCS cognitive response direction	Plewnia et al. (2015)
Neurophysiological	Baseline cortical excitability	Response depends on pre-stimulation E/I balance	Silvanto and Pascual-Leone (2008)
	Spontaneous oscillatory state	Ongoing EEG phase modulates plasticity induction	Zrenner et al. (2018)
Methodological	Current density (E-field at cortex)	Higher cortical E-field drives stronger cognitive and motor effects	Caulfield et al. (2022) and Razza et al. (2024)
	Electrode size and position	Affects spatial distribution, focality, and edge-current patterns	Miranda et al. (2006) and Niemann et al. (2024)
	Gel conductivity	High-conductivity EEG gel increases current spread	Saturnino et al. (2015)
	Online vs. offline timing	Online stimulation during task amplifies state-dependent effects	Silvanto and Pascual-Leone (2008)

### Paths to personalization

**Figure 2:**
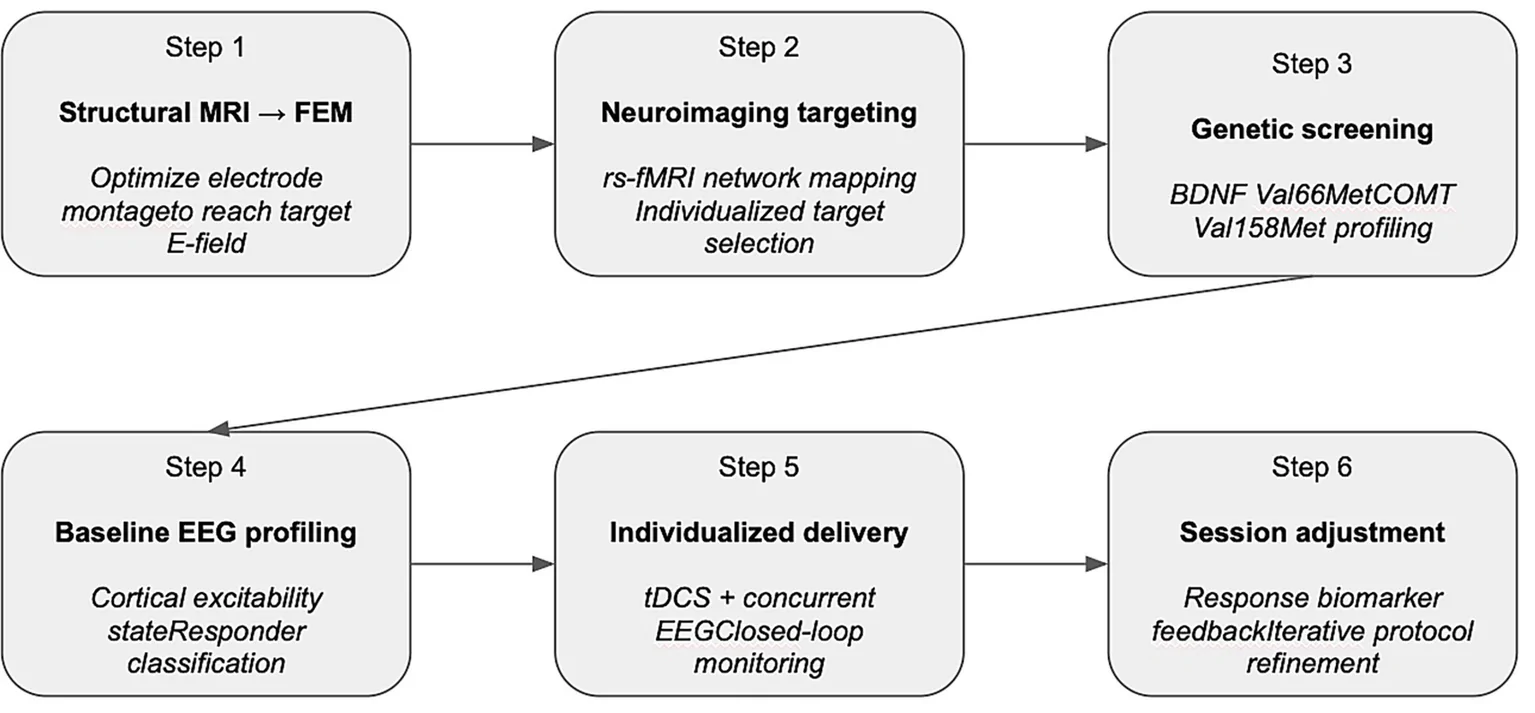
A stepwise personalization pipeline for individualized tDCS protocol design.

Several methodological approaches have been proposed to bring tDCS protocols closer to individual biology. They operate at different points in the stimulation workflow, from pre-stimulation modeling to real-time adaptive delivery, and are not mutually exclusive. Figure 2 outlines a stepwise personalization pipeline that integrates these approaches into a unified sequence.

### Computational electric field modeling

Subject-specific FEM modeling from structural MRI is the most established individualized approach. By building a computational head model for a given participant, researchers can estimate the electric field distribution in that person’s brain before delivering any current, then optimize electrode placement, size, and intensity to hit a target field strength at the intended site (Bikson et al., 2016; Truong et al., 2013).

Several studies have provided direct evidence that this approach works. Van Hoornweder et al. (2022) demonstrated a prospective E-field dosing method (2-SPED) across 300 participants using three common montages, showing that individualized intensity prescriptions substantially reduced E-field variance at motor cortex targets compared to uniform-dose protocols (Van Hoornweder et al., 2022). For cognitive outcomes, Caulfield et al. (2022) found that higher individual E-field strength at the left DLPFC under uniform 2 mA dosing predicted working memory improvements, and that reverse-calculation individualized dosing significantly reduced variance in the cortical E-field (Caulfield et al., 2022).

MRI-guided electrode placement reduces variability in target engagement compared to standard 10–20 system positioning (Opitz et al., 2016; Miranda et al., 2013). Automated pipelines—SimNIBS, ROAST - have made subject-specific modeling considerably less burdensome, bringing it within reach of research settings and, eventually, clinical use (Thielscher et al., 2015).

Van der Cruijsen et al. (2022) applied this logic directly to stroke patients, showing that patient-tailored FEM montages produced on average 20% stronger fields at the motor target—with individual improvements up to 52%—than conventional montages (van der Cruijsen et al., 2022). Their results specifically support the use of individualized models in clinical stroke rehabilitation, where lesion anatomy makes group-derived montages especially unreliable.

### Neuroimaging-guided targeting

Anatomical modeling aside, functional and structural neuroimaging can refine target selection based on individual neural correlates of the intended outcome. For cognitive applications, resting-state fMRI connectivity can identify which network nodes are most strongly linked to the target function—default mode network for memory, frontoparietal network for attention—and electrode placement can be optimized around each participant’s actual topology rather than group-average coordinates.

In neurodegenerative conditions this matters especially. Bhattacharjee et al. (2024) simulated tDCS in 50 dementia patients and found that cortical atrophy caused the individual E-field at target regions to be lower and more variable than in healthy controls, requiring higher personalized doses. For HD-tDCS with fixed electrode positions, 46% of dementia patients would have required currents exceeding the 4 mA safety limit to achieve adequate target stimulation—a finding that directly argues against applying healthy-population fixed-montage protocols in this population (Bhattacharjee et al., 2024).

### Genetic profiling for stratification

Genetic polymorphisms in BDNF and COMT reliably predict tDCS response direction and magnitude. Including genotype as a stratification variable—either by balancing enrollment or by including it as a covariate—would reduce biologically driven variability and increase statistical power. This is pharmacogenomics for brain stimulation. Given the falling cost of genotyping, it is actually near-term feasible.

### Closed-loop and adaptive stimulation

Closed-loop systems are the most technically demanding personalization approach. Rather than delivering a predetermined current regardless of what the brain is doing, closed-loop tDCS uses EEG or other neural signals to trigger or adjust stimulation when the brain is in a state receptive to neuromodulation (Zrenner et al., 2018).

A proof-of-concept study by Caravati et al. (2024) examined closed-loop tDCS that dynamically adapted both current intensity and stimulation site based on real-time EEG entropy metrics (approximate entropy, sample entropy) during a sustained attention task in healthy students. Adaptive tDCS significantly improved both accuracy (94.04% vs. 90.82%) and reaction times (262.93 ms vs. 302.03 ms) compared to a non-stimulation condition, with stimulation decisions driven by the individual’s instantaneous neural state rather than a fixed schedule (Caravati et al., 2024). This represents an early but concrete demonstration that state-responsive tDCS delivery is technically feasible.

### Toward a standardized personalization pipeline

A practical personalization sequence might run as follows: (1) structural MRI to generate a subject-specific FEM model; (2) electrode montage optimization to achieve target field strength; (3) optional functional neuroimaging to refine target selection; (4) genetic screening for BDNF Val66Met and COMT Val158Met; (5) baseline EEG characterization of cortical excitability; (6) tDCS delivery with concurrent neurophysiological monitoring; and (7) iterative protocol adjustment based on response biomarkers.

The full pipeline is resource-intensive and currently limited to specialized research settings. But adopting individual components, FEM-guided montage design especially, is achievable now and would move practice meaningfully forward compared to the uniform-protocol default.

## Conclusion

Three categories of factors reliably produce inconsistent tDCS outcomes. Biological: individual neuroanatomy, cortical thickness, gyrification pattern, CSF layer thickness, and genetic polymorphisms in BDNF and COMT. Neurophysiological: baseline excitability state, spontaneous oscillatory activity, excitation-inhibition balance, and the state-dependency of neuromodulatory effects. Methodological: electrode montage, current density, stimulation duration, gel conductivity, electrode positioning errors, and the absence of reporting standards that would make study-to-study comparison meaningful. None of these is minor, and none operates in isolation.

Recent work has made the quantitative cost of these sources of variability concrete. Inter-individual differences in current direction, not just magnitude, across common montages range from 50 to 150% (Evans et al., 2022). Electrode positioning deviations in focal set-ups reduce target current by 26–30% (Niemann et al., 2024). Individual E-field strength at the prefrontal cortex, not scalp dose, predicts working memory improvement from tDCS (Caulfield et al., 2022; Razza et al., 2024). Meta-analyses in both AD and PD find no significant pooled effects, yet meta-regressions consistently reveal subgroups—younger PD patients, patients receiving combined tDCS plus training—that do respond, pointing to uncontrolled individual-level moderators as the primary source of null average effects.

In rehabilitation for Alzheimer’s and Parkinson’s diseases, these sources of variability are compounded by the neuroanatomical changes of neurodegeneration itself. Cortical atrophy alters current flow patterns in ways that are highly individual. Protocols developed on healthy young adults transfer poorly to these populations. Subject-specific FEM modeling and individualized target selection are necessary here, not optional add-ons—and both are more feasible now than they were 5 years ago.

FEM modeling, neuroimaging-based target selection, genetic stratification, and closed-loop stimulation each address a different layer of the variability problem. Used together, they add up to something resembling precision neuromodulation. Their sequential integration into a personalization pipeline is technically feasible and would advance reproducibility faster than any single methodological fix.

The most useful near-term research priorities are: computational modeling as a minimum study design standard; genetic covariate inclusion (BDNF Val66Met and COMT Val158Met, at minimum) in clinical trials; larger pre-registered RCTs with stratified enrollment; EEG-based biomarker validation for real-time responder classification; and combined behavioral-stimulation paradigms built around state-dependency principles. Without progress on these fronts, tDCS will remain what it largely is now: interesting in a lab, unreliable in a clinic.
