# Correction: The inconsistent effects of tDCS in rehabilitation and cognitive enhancement: sources of variability and paths to personalization

**DOI:** 10.3389/fnhum.2026.1980636

**Published:** 2026-09-15

**Authors:** Andrey Timashkov, Sergey Andreev, Anna Safonova, Samira Zangieva, Dzerassa Kadieva, Oksana Zinchenko

**Affiliations:** 1 Centre for Cognition and Decision Making, Institute for Cognitive Neuroscience, HSE University, Moscow, Russia; 2 Faculty of Social Sciences, School of Psychology, HSE University, Moscow, Russia

**Keywords:** Alzheimer's disease, cognitive enhancement, inter-individual variability, Parkinson's disease, rehabilitation, tDCS, transcranial direct current stimulation

An incorrect text was provided for the **Funding** statement. It previously stated: “The author(s) declared that financial support was received for this work and/or its publication. The publication was prepared within the framework of the Academic Fund Program at HSE University (grant No 24-00-008 “Neural correlates of neurodegenerative disorders”).” The correct text is “The author(s) declared that financial support was received for this work and/or its publication. This article is an output of a research project HSE-BR-2025-26 implemented an output of the Basic Research Program at HSE University.”

The original version of this article has been updated.

